# The Molecular Basis and Pathophysiology of Trigeminal Neuralgia

**DOI:** 10.3390/ijms23073604

**Published:** 2022-03-25

**Authors:** QiLiang Chen, Dae Ik Yi, Josiah Nathan Joco Perez, Monica Liu, Steven D. Chang, Meredith J. Barad, Michael Lim, Xiang Qian

**Affiliations:** 1 Department of Anesthesiology, Perioperative and Pain Medicine, Stanford University School of Medicine, Stanford, CA 94305, USA; chenqi@stanford.edu (Q.C.); daeikyi@stanford.edu (D.I.Y.); josiahp@stanford.edu (J.N.J.P.); monicaml@stanford.edu (M.L.); mbarad@stanford.edu (M.J.B.); 2 Department of Neurosurgery, Stanford University School of Medicine, Stanford, CA 94305, USA; sdchang@stanford.edu (S.D.C.); mklim@stanford.edu (M.L.)

**Keywords:** trigeminal neuralgia, pathophysiology, sensitization, classifications, treatments

## Abstract

Trigeminal neuralgia (TN) is a complex orofacial pain syndrome characterized by the paroxysmal onset of pain attacks in the trigeminal distribution. The underlying mechanism for this debilitating condition is still not clearly understood. Decades of basic and clinical evidence support the demyelination hypothesis, where demyelination along the trigeminal afferent pathway is a major driver for TN pathogenesis and pathophysiology. Such pathological demyelination can be triggered by physical compression of the trigeminal ganglion or another primary demyelinating disease, such as multiple sclerosis. Further examination of TN patients and animal models has revealed significant molecular changes, channelopathies, and electrophysiological abnormalities in the affected trigeminal nerve. Interestingly, recent electrophysiological recordings and advanced functional neuroimaging data have shed new light on the global structural changes and the altered connectivity in the central pain-related circuits in TN patients. The current article aims to review the latest findings on the pathophysiology of TN and cross-examining them with the current surgical and pharmacologic management for TN patients. Understanding the underlying biology of TN could help scientists and clinicians to identify novel targets and improve treatments for this complex, debilitating disease.

## 1. Introduction

Trigeminal neuralgia (TN), previously known as tic douloureux, is a chronic neuropathic pain syndrome characterized by recurrent unilateral lancinating facial pain limited to the distribution of the trigeminal nerve dermatome [1]. It is a debilitating condition with severe pain and an unpredictable course that negatively impacts patients [2,3,4]. Patients with TN are often anxious about pain episodes, which discourages them from performing basic daily routines such as talking, eating, brushing teeth, and even participating in social activities. Overall, patients with TN undergo a significant amount of stress, which may lead to anxiety and depression, resulting in poor quality of life [5,6,7,8,9,10].

Traditionally, TN has been a clinical diagnosis, based on patient-reported symptoms [11]. However, advancement in clinical and basic science research has led to a deeper understanding of TN anatomy, pathophysiology, and symptomatology, which has helped to delineate the different subtypes of the disease. Most recently, the International Headache Society (IHS) and International Association for the Study of Pain (IASP) established a new classification system for TN, which includes subtypes of classical TN, secondary TN, and idiopathic TN [1]. This classification scheme aims to incorporate the current understanding of the pathophysiology of the disease with decades of clinical experiences to better aid physicians in effectively diagnosing TN subtypes and thereby guiding treatments based on the diagnosis.

The current article aims to review our current understanding of the basic mechanism of the disease and cross-examine these new data with the disease symptomatology and the current management strategies. Lastly, the outlook for future research and clinical practice will also be briefly discussed.

## 2. Clinical Features

### 2.1. Epidemiology

The recurring, episodic, and unpredictable clinical course of TN makes it difficult to determine the exact incidence of the disease. The estimated incidence of TN ranges from 4.3 to 26.8 per 100,000 person-years, with a lifetime prevalence of 0.03% to 0.3% [2,3,4,12,13]. Although the causes are not fully elucidated, the incidence of TN in women is higher than men, and the average age of onset is 53, with the most affected ages between 37 and 67 [3,12,13,14,15]. Most cases of TN are sporadic without apparent risk factors. However, there have been a few reports of familial TN [16,17], which have led to investigations of the possible genetic and molecular basis to the pathophysiology of TN.

### 2.2. Symptomology

TN can be debilitating because it causes a significant amount of pain in the orofacial region that is often disabling to the patient. The paroxysmal electric or stabbing pain attacks are transient and episodic, lasting less than 10 to 15 s [14,18,19]. These attacks are usually unilateral, although there are rare cases where the bilateral trigeminal nerves are involved [4,14,20]. The most affected branches of the trigeminal nerves are the maxillary (V2) and the mandibular (V3) branch, or a combination of the two, with the ophthalmic branch (V1) rarely affected [4,15,21]. A refractory phase of the pain-free period will follow after a series of pain attacks, although the length of the refractory period varies amongst patients [14]. Interestingly, recent studies revealed that 30 to 49% of patients endorsed a concomitant continuous dull, throbbing, and burning pain, with an onset of 1.5 years after the initial symptoms [15,19].

One of the major reasons why TN is debilitating is that normal daily activities can trigger severe pain. The most frequent triggering factors were touching the face, talking, chewing, or brushing teeth [5,10]. Although TN is known to be precipitated by innocuous stimuli, a study showed that a few percent of patients reported unusual trigger maneuvers, including flexing the trunk, contact with hot or cold food/water, speaking loudly, and turning of the eyes [5]. About a third of patients experienced autonomic symptoms, such as conjunctival tearing or injection on the ipsilateral side during TN attacks [14,18]. Other symptoms include edema and local flushing in the distribution of the trigeminal nerves that are affected [19]. Unsurprisingly, TN can impose significant psychosocial stress on patients and negatively impact their quality of life. Many patients suffering from TN endorse higher rates of anxiety and depression [6,9].

### 2.3. Clinical Classifications

TN-related symptoms have been described in historical documents as early as in the sixteenth century [22]. It was not until the late 20th century that the disease was further subclassified based on its attack characteristics into typical TN and atypical TN [23,24]. Typical TN was described as sharp, electrical, paroxysmal, and primarily located in the V2 and V3 regions of the trigeminal nerve, whereas atypical TN was dull, constant, and located in all three divisions (Table 1). However, it was challenging to guide treatment based on this classification scheme. Fortunately, the classification of TN has since evolved as our understanding of TN also broadened significantly with scientific advances within the past decades.

The rapid technological development of magnetic resonance imaging (MRI) has helped identify some of the appreciable anatomical changes in TN, which facilitated the new classification scheme [25]. High-resolution images revealed that neurovascular compression at the trigeminal nerve root entry zone correlates strongly with TN symptoms and anatomical nerve changes such as nerve atrophy, dislocation, indentation, or flattening [26,27]. Similar anatomical changes are also observed in TN associated with other primary demyelinating disorders [28,29,30,31]. To create a classification of TN that is more universally accepted among clinicians and academics, both the International Headache Society (IHS) and the International Association for the Study of Pain (IASP) incorporated these observations and published new classifications in 2018 [1,32,33]. They currently describe TN as a disorder presented with recurrent abrupt-onset unilateral brief electric shock-like pains triggered by innocuous stimuli that limit the distribution of one or more divisions of the trigeminal nerve [1]. They further classified TN into three subgroups (e.g., classical, secondary, and idiopathic TN) based on anatomical and electrophysiological findings (Table 1).

In addition to the IHS and IASP classifications, other groups also independently published classifications of TN-related facial pain. For example, Burchiel and colleagues classified facial pain syndromes into seven categories to help develop a framework to better diagnose and treat different types of TN based on the pain characteristic or the inciting event. Burchiel’s classification includes TN type I (sharp, electrical shock-like, episodic pain due to neurovascular compression of TN), TN type II (aching, throbbing, burning, constant pain >50% of the time), TN due to injury (e.g., trauma, post-surgical pain), TN secondary to multiple sclerosis, infection-related postherpetic TN, and atypical somatoform facial pain [34,35]. This unique classification scheme aims to personalize treatment recommendations based on accurate patient history, although further studies are required to verify its clinical utility.

## 3. Pathophysiology of Trigeminal Neuralgia

### 3.1. Compression of the Trigeminal Nerve Root

Classical TN is defined by focal neurovascular compression of the trigeminal nerve structure, usually occurring at the junction of the peripheral trigeminal nerve and root by vasculatures at the prepontine cistern within the Meckel’s cave [1,36]. While direct contact with arteries or veins on the trigeminal nerve root is the most common compressive mechanism, other space-occupying lesions, such as arteriovenous malformation, aneurysm, vestibular schwannoma, meningioma, and other types of cysts and tumors can also lead to trigeminal nerve compression [1,27,37,38,39,40,41]. While direct compression on the trigeminal nerve root has long been hypothesized to be the primary trigger for classical TN, the cascade of reactions that follows and the fundamental mechanism through which they lead to TN symptoms are still not well understood.

Histological examinations of the trigeminal nerve in classical TN patients have provided clues on the pathogenesis of the disease. Compression of the trigeminal nerve is associated with a significant level of myelin erosion and disintegration from inflammation, particularly at the nerve indentation area [42,43,44,45,46]. This structural abnormality is related to pathologic demyelination and remyelination of the injured nerve, a common feature in humans with peripheral nerve compression and animal models of chronic nerve compression [47,48,49,50,51]. Furthermore, Devor and colleagues reported Schmidt-Lanterman incisures in trigeminal nerve root biopsies from TN patients, consistent with a pathologic increase in metabolic demand for myelin sheath growth and maintenance found in chronic nerve compression [52,53]. Finally, there is evidence of axonal dystrophy and Schwann cell damage associated with trigeminal nerve compression [45,51,54,55,56].

Subsequently, these compression-related structural changes trigger several crucial downstream effects that play an important role in developing TN symptoms. Dysregulation of voltage-gated sodium (Nav) channels is functionally linked to TN [57]. Both preclinical animal models of classical TN and biopsies from TN patients showed a significant upregulation of Nav1.3 [58,59,60]. While Nav1.3 is an embryotic channel type normally suppressed in adults, its overexpression has been associated with several neuropathic pain conditions [61,62]. Electrophysiological recording of Nav1.3 demonstrated rapid and persistent channel activations in response to electrical stimulation [63]. This unique channel mechanic of Nav1.3 could be a major contributor to the enhanced sensitivity and ectopic impulse generation in a compressed trigeminal nerve.

In contrast, downregulation of Nav1.7 in the trigeminal nerve root is found in TN patients and preclinical TN models [58,59,60]. Nav1.7’s channel mechanic is characterized by fast inactivation and slow recovery, making it resistant to repetitive action potentials [64]. Furthermore, Nav1.7 can respond to graded potentials while in its prolonged close-gated inactivated state, which functionally translates it into a threshold channel [64]. Therefore, decreased Nav1.7 expression in the context of Nav1.3 upregulation could further increase neuronal excitability and impair normal nociceptive responses in TN. Upregulation of other Nav channels, such as Nav1.1, has recently been shown to associate with the hyperactivity of the trigeminal nerve in a chronic constricted nerve injury model in rodents [65]. However, the precise mechanism of these Nav channel dysregulations and dysfunctions contributing to symptomatic TN need to be further characterized. Additionally, hyperexcitability in trigeminal neurons secondary to the dysregulation of the resting potential mediated by the voltage-gated potassium channel has been identified in preclinical models of classical TN [66]. Lastly, Gupta and colleagues demonstrated significant apoptosis and persistent downregulation of myelin-associated glycoprotein in Schwann cells following chronic nerve compression injury [49,67]. Given Schwann cells’ ability to inhibit axonal growth via their expression of myelin-associated glycoprotein, loss of such intrinsic growth regulation could lead to the axonal sprouting seen in classical TN [45,51,54,55,68]. Indeed, this compression-induced axonal pathology provides the mechanistic basis for nerve regeneration techniques, such as type-I collagen implantation, as a potential treatment for TN [69].

The channelopathies and pathologic changes in the architectures of afferent neurons subsequently result in functional hyperexcitability of the trigeminal nerve observed in TN. Indeed, recording of trigeminal nerve roots in models of classical TN demonstrated ectopic generations of action potentials and prolonged after-discharges in demyelinated neurons [70,71,72,73]. These ectopic discharges are thought to be further amplified and spread through ephaptic cross-talking between demyelinated fibers, even between functionally distinctive neurons (e.g., A-beta afferents and nociceptive C-fibers) [74,75,76,77]. Taking these data together, Devor and colleagues proposed the “ignition hypothesis” to provide a pathophysiological explanation for the clinical characteristics of classical TN [78]. Briefly, demyelination and hyperexcitability of trigeminal neurons following neurovascular compression decrease the triggering threshold for activation of sensory afferents. During a paroxysmal attack in TN, a normally innocuous stimulus on the triggering area would lead to amplified trigeminal afferent inputs by the means of prolonged discharges and ephaptic cross-talks between the demyelinated axons. Such an attack could slowly subside if the offending stimulus is removed or the triggering threshold is raised, such as through pharmacological blockade of dysfunctional channels or functional recovery after vascular decompression surgery [78].

### 3.2. Primary Demyelinating Diseases

Although neurovascular compression accounts for most TN cases, primary demyelination disorders, such as multiple sclerosis, can also lead to TN symptoms. It has been well documented that patients with MS are at a higher risk of developing neuropathic pain and are estimated to be twenty times more likely to develop TN than the general population [79,80]. Pathological examinations and radiological evidence from MS patients with TN have demonstrated significant inflammatory demyelination at the trigeminal nerve root [28,29,30,81,82]. Concurrent neurovascular compression on the trigeminal nerve in MS patients could accelerate demyelination through both mechanical and inflammatory mechanisms, which lead to TN symptoms [83,84]. These observations lead to the theory of demyelinating plaque formation at the trigeminal nerve root being the main cause of TN symptoms in MS patients through a similar mechanism as in compression-related TN [29,83].

However, it has also been speculated that central demyelinating lesions can also independently lead to TN. Intrapontine demyelination along the trigeminal afferent and the trigeminal nucleus has been associated with TN symptoms in patients with MS or a brainstem infarction [31,85,86,87,88,89,90]. Although neurovascular compression is occasionally found concurrently in these patients [83,84], demyelination of the primary trigeminal afferent intrapontine trigeminal nucleus alone is sufficient to lead to TN [91]. Furthermore, at least a subset of these patients with comorbid TN and MS had lesions in second-order sensory neurons in the brainstem ipsilateral to the affected side, which are thought to cause trigeminal pain and other facial sensory disturbances [29,92].

### 3.3. Sensitization and Dysfunction of Central Pain-Related Circuits

Central sensitization is a process through which the nociceptive system becomes hyperexcitable or a state of hyperexcitability, and it has been implicated in various chronic pain conditions [93,94]. With the advancement of electrophysiological and advanced functional imaging techniques, researchers are now able to closely examine the sensitization of central nociceptive and affective processing in patients with classical or idiopathic TN.

Amplified nociceptive signal transmission is found in TN patients. In a series of electrophysiological recordings in TN patients with concomitant chronic facial pain, Obermann and colleagues demonstrated that pain-related evoked potentials are significantly augmented in all trigeminal divisions on both symptomatic and non-symptomatic sides [95]. This finding suggests that sensitization of the trigeminal pathway as well as the supraspinal pain-modulating circuits may be an important part of TN pathophysiology.

Functional imaging data in TN patients has also helped further delineate the involvement of pain-related supraspinal structures. Unsurprisingly, painful attacks in TN patients lead to increased activity in the trigeminal nuclei, thalamus, and somatosensory cortices—areas that are classically associated with pain-related sensory processing [96]. Moreover, multiple vital structures related to pain modulation, emotion, and memory are also activated during the attacks. These structures include the anterior cingulate cortex, insula cortex, prefrontal cortex, hippocampus, limbic system, and the brainstem pain-modulation system [96,97]. Sensitization in some of these structures has been implicated in other chronic pain conditions [93,94,98,99]. Furthermore, structural and functional neuroimaging data revealed significant alterations in functional connectivity of the frontal-limbic circuit and a gray matter reduction in pain-modulating, sensory-motor, and affective circuits in TN patients compared with healthy subjects [97,100]. Interestingly, these pathological changes are often reversed after successful treatments, suggesting changes in these circuits not only link to the characteristic pain symptoms but also the psychocognitive aspect of the disease [96,97].

## 4. Treatment

### 4.1. Medical Therapies

#### 4.1.1. Maintenance Therapy

Anticonvulsant medications form the mainstay of medical therapies for TN. Of these, the best evidence exists for carbamazepine, which has been found to be effective in multiple randomized controlled trials [101,102,103]. Oxcarbazepine, a structural analog of carbamazepine, is also considered an effective first-line medical treatment [104]. Targeting the channelopathy seen in TN, carbamazepine and oxcarbazepine are both Nav blockers aiming to stabilize hyperexcited neuronal membranes and reduce ectopic nociceptive signaling. Despite their effectiveness, the use of both medications is often limited by their side effects, which include drowsiness, dizziness, rash, ataxia, elevated liver enzymes, hematologic dyscrasias, and hyponatremia. A recent study demonstrated that oxcarbazepine might be better tolerated than carbamazepine, although any side effects may be seen with either medication [105].

If the first-line anticonvulsants are ineffective or poorly tolerated, other anticonvulsive agents, including lamotrigine, gabapentin, and pregabalin, can also be considered as second agents or as monotherapy [104]. Lamotrigine, another Nav blocker, has demonstrated an analgesic effect in a small group of patients with refractory TN when added as a second agent [106]. The evidence for using gabapentin, a voltage-gated calcium channel blocker, in TN was summarized in a meta-analysis comparing it with carbamazepine [107]. With sixteen Chinese studies included, this meta-analysis provides low- to moderate-quality evidence that gabapentin may be as effective as carbamazepine while generally being better tolerated with fewer side effects [107]

Trigger point injections have also been explored as a maintenance strategy. For example, when used in conjunction with gabapentin, injection of ropivacaine, a local anesthetic, into facial TN pain trigger points has been shown to provide lasting pain relief for at least 28 days [108]. Multiple randomized controlled trials have demonstrated that injection of botulinum toxin A, a neurotoxin derived from the bacteria *Clostridium botulinum*, is an effective maintenance treatment for TN [109,110,111,112]. By injecting the toxin directly into the trigger points, it is thought to produce a lasting analgesic effect via lesioning the hyperactive fibers in the affected trigeminal branches [112,113]. Single treatments have been shown to significantly improve anxiety, depression, sleep, pain, and the number of attacks per day for up to 12 weeks [111,114]. However, due to its neurotoxic and paralytic effect, patients often report facial asymmetry with dynamic movement and facial edema after botulinum toxin injections [111].

Lastly, manual acupuncture and electroacupuncture, based on traditional Chinese medicine concepts, could be effective adjunct therapies for TN [115]. It was hypothesized that stimulation of peripheral acupuncture points leads to central nociceptive modulation and upregulation of the endogenous opioid system [115,116]. However, it is important to note that high-quality clinical evidence supporting these techniques is still lacking. A recent meta-analysis of 33 randomized controlled trials in China found mixed results for the use of acupuncture in TN [117]. Most of the trials included in the meta-analysis found that acupuncture techniques effectively reduce TN pain attack intensity and recurrence rate and may even be synergistic when combined with carbamazepine, an anticonvulsant [117]. However, the authors caution against the widespread use of acupuncture for TN due to the subpar quality of the currently available trials [117]. Indeed, the clinical application of acupuncture outside of China remains controversial. Most clinical trials are limited by their small sample size, non-blinding design, short follow-up period, and non-standardized measurement of outcomes. Furthermore, critics of acupuncture argue that trials conducted in China might have an inherent cultural bias favoring the technique and could be difficult to control [115]. Thus, establishing reliable basic science models for acupuncture and conducting large-scale multi-region trials with standardized outcome measurements and long-term follow-up could help delineate the mechanism and better characterize the therapeutic effects of acupuncture as a treatment for TN.

#### 4.1.2. Abortive Therapies

In addition to maintenance therapies, other drugs have been studied for use as abortive treatment during acute pain attacks. An intranasal spray of 8% lidocaine has also shown statistically significant pain reduction for four hours in patients with second-division TN [118]. Intravenous phenytoin, yet another Nav blocker, demonstrated an acute response rate of 89% in a retrospective cohort, including classic, idiopathic, and secondary TN [119]. Fosphenytoin, a prodrug of phenytoin, has similarly shown positive results in aborting TN exacerbations in small case reports [120,121].

### 4.2. Procedural Interventions

#### 4.2.1. Neurological Surgeries

Microvascular decompression (MVD) is considered the first-line surgical procedure for patients with clear neurovascular compression etiology determined by imaging [122,123]. It has a well-established record of a favorable outcome, with an approximately 70% pain-free rate after the first two years post-surgery [123,124,125]. Surgical technique is also being improved over time. In a recent prospective cohort study, Mizobuchi and colleagues demonstrated complete pain relief in 80% of patients at three years follow-up, with their higher success rate attributed to transposing the causative vessel away from the trigeminal nerve with a prosthesis, rather than interposing a prosthesis between the vessel and nerve [126]. The authors in the same study also observed that an arterial compressive pattern better predicted a successful response when compared to patterns of compression due to venous structures or arachnoiditis [126].

For those without neurovascular compressions, particularly when MVD is initially intended but compression is not observed intraoperatively, open interfascicular neurolysis can be considered [127]. This technique aims to induce micro-trauma in the hyperactive trigeminal nerve by dividing the nerve longitudinally into multiple fascicles, which is thought to interrupt abnormal nociceptive transmissions and induce remyelination in the trigeminal system [127,128,129]. Although multiple retrospective studies have shown long-term symptom relief in 70–90% of the patients by this technique, the broad application of open neurolysis is limited by its invasive nature, along with the retrospective design and the small sample size of the supporting clinical studies [127,129,130,131].

Additional to the open surgical approaches, minimally invasive stereotactic radiosurgery (SRS), such as Gamma Knife and Cyberknife, which involves the application of ionizing radiation to the trigeminal nerve, is a viable alternative therapeutic option for TN. The exact mechanism of pain relief in SRS is unknown. Still, it is thought that targeted ionizing radiation damages sodium channels on the trigeminal fiber, which interrupts afferent sensory transmission [132]. Although SRS is less effective in producing lasting pain relief than MVD, it has been shown to be associated with fewer complications [133,134,135]. It also appears that SRS can be safely repeated for recurrent TN pain [136].

#### 4.2.2. Percutaneous Techniques

When a patient cannot tolerate neurological surgery or presents without underlying vascular compression, percutaneous interventions can be offered. These techniques can be subclassified into radiofrequency thermal ablation, balloon compression, and chemical rhizotomy.

Percutaneous thermal rhizotomy with radiofrequency ablation (RFA) was first introduced by Sweet and colleagues. They hypothesized that carefully graded increments of heat could selectively damage smaller myelinated and unmyelinated fibers that are responsible for pain transmission, since the non-nociceptive A-beta fibers are relatively protected from heat due to their heavier myelin sheaths [137]. They reported a 91% rate of immediate pain relief, with a recurrence rate of 22% in patients followed over 2–6 years [137]. This finding was later confirmed by a retrospective cohort analysis of 1000 consecutive patients who underwent RFA, which showed a 94.8% immediate response rate, allowing for discontinuation of medical treatment, with an 18% recurrence rate over an average follow-up of 9 years [138]. However, 20% of patients in that study developed corneal reflex impairment, with six patients having keratitis that required surgery due to either tarsorrhaphy or enucleation [138]. RFA is also associated with an increased risk of pain recurrence and postoperative facial anesthesia compared to MVD [139]. Interestingly, it has been suggested that RFA is equally effective for patients with and without neurovascular compression, making it a viable option for those patients who are not candidates for MVD [140].

Percutaneous balloon compression (PBC) is another technique that aims to treat TN by lesioning afferent fibers at the level of the Gasserian ganglion. A recent retrospective study suggested a first-time success rate of 89% after PBC, with 76% of patients able to titrate off pain medicines [141]. Data from the past two decades support PBC as an effective therapy by showing it to be equally effective as other techniques [104,123,124,142,143,144]. Unfortunately, PBC is associated with an inconsistent recurrence rate, ranging from 19 to 59%, although repeat procedures do appear to be nearly equally efficacious [141,145]

Lastly, percutaneous glycerol rhizotomy relies on the chemical ablation of pain-transducing nerve fibers. It carries a similar success rate to other percutaneous techniques but can have a recurrence rate of up to 50% by three years post-procedure [146]. Similarly, repeat procedures have also been safe and effective [147].

## 5. Conclusions and Future Directions

In the past three decades, significant progress has been made to understand the pathophysiology of TN. The demyelination-induced trigeminal hyperexcitability theory has offered a critical mechanistic basis for the diagnosis and the implementation of current medical therapies (e.g., anticonvulsants, antidepressants), surgical procedures (e.g., MVD, Cyberknife, and Gamma Knife), and percutaneous techniques (e.g., RFA, PBC, glycerol rhizotomy) for TN. However, several crucial observations related to disease classification, diagnosis, and treatment efficacy have left some aspects in the current explanation of TN pathophysiology rather unsatisfying.

It has been increasingly recognized that TN’s etiology is likely multifactorial in many patients. Only a small percentage of TN patients present with demonstrable compression or morphological changes in the trigeminal nerve, and neurovascular compression does not always translate to disease [27,148,149]. Furthermore, most patients present with a normal physical and neurological exam, and there is a lack of reliable biomarkers for the disease [14,55,150,151]. Thus, such complex disease presentation makes accurate diagnosis of TN difficult for some patients and may even challenge the utility of the current classification scheme [152]. Lastly, TN often recurs with increasing resistance to therapies over time [153]. Conventionally, procedural interventions will be offered when medical therapies fail, but there is little data to guide decisions on the timing of this decision. Even though, based on a few studies, surgical and minimally invasive interventions appear to have a promising track record in providing relief, they are also known to accompany a varying degree of complications and recurrence [151].

Therefore, the lack of complete understanding of TN’s complex etiology, pathogenesis, and pathophysiology poses a challenge for clinicians and basic scientists in discovering targeted therapies for patients. Future studies focusing on investigating the basic mechanism for TN, such as genetics, molecular biology, electrophysiology, and functional imaging could further improve the precision of diagnosis and therapies for patients suffering from this debilitating disease.

## Figures and Tables

**Table 1:** Comparison of TN Classifications.

Classifications	ICHD-3/IASP	Typical vs. Atypical TN by Rasmussen	Burchiel Classifications
Characteristic	The classification was developed based on consensus between the International Headache Society (IHS) and the International Association for the Study of Pain (IASP) to create a classification of TN that is more universally accepted among clinicians and academics.	The first classification that attempted to further subclassify TN based on its attack characteristics in 1990	The classification by Burchiel et al. categorized seven types of TN based on the pain characteristic or its associated eliciting event in order to provide a framework to better diagnose and treat different types of TN.
Subclassifications	- Classical TN *—typical symptomatic TN due to neurovascular compression of trigeminal nerve evidenced by imaging (MRI) or surgery - Secondary TN—typical symptomatic TN caused by an underlying disease, other than the neurovascular compression, which includes Multiple Sclerosis, space-occupying lesions including cerebellopontine angle, AV malformation or fistula, skull-base bone deformity, connective tissue disease, and genetic causes of neuropathy - Idiopathic TN *—symptomatic TN with neither MRI nor electrophysiological tests revealing significant abnormalities, suggesting TN without obvious or visible etiologies that are not fully understood yet * Classical and Idiopathic TN are also sub-categorized as “purely paroxysmal” or “with concomitant continuous pain” in an effort to address the timing of pain attacks.	- Typical TN: pain described as sharp, electrical, paroxysmal, and mostly located in the V2 and V3 regions of the trigeminal nerve - Atypical TN: pain described as dull, constant, and located in all 3 divisions	- TN type I—sharp, electrical shock-like, episodic pain due to neurovascular compression of TN - TN type II—aching, throbbing, burning, constant pain >50% of the time - TN due to injury—Trigeminal neuropathic pain (unintentional) e.g., facial trauma - Trigeminal deafferentation pain (intentional) e.g., post-surgical - TN secondary to multiple sclerosis - Infection-related postherpetic TN - Atypical somatoform facial pain
Comments	- Most recent classification for TN (published in 2018) - Requires imaging and/or electrophysiological findings for subclassification diagnosis - Helps guide further treatment modalities (medical vs. surgical)	- This classification was too broad to guide specific treatment based on symptoms alone.	- This classification attempts to guide differential diagnosis by using objective and reproducible criteria. - Requires further studies to verify clinical utility

## Data Availability

Not applicable.
